# Effect of cyclooxygenase inhibition on cholesterol efflux proteins and atheromatous foam cell transformation in THP-1 human macrophages: a possible mechanism for increased cardiovascular risk

**DOI:** 10.1186/ar2109

**Published:** 2007-01-23

**Authors:** Edwin SL Chan, Hongwei Zhang, Patricia Fernandez, Sari D Edelman, Michael H Pillinger, Louis Ragolia, Thomas Palaia, Steven Carsons, Allison B Reiss

**Affiliations:** 1 Division of Clinical Pharmacology, Department of Medicine, New York University School of Medicine, 550 First Avenue, New York, NY 10016, USA; 2 Vascular Biology Institute, Department of Medicine Winthrop-University Hospital, 222 Station Plaza, North, Mineola, NY 11501, USA; 3 Division of Rheumatology, Allergy and Immunology, Department of Medicine Winthrop-University Hospital, 222 Station Plaza, North, Mineola, NY 11501, USA

## Abstract

Both selective cyclooxygenase (COX)-2 inhibitors and non-steroidal anti-inflammatory drugs (NSAIDs) have been beneficial pharmacological agents for many patients suffering from arthritis pain and inflammation. However, selective COX-2 inhibitors and traditional NSAIDs are both associated with heightened risk of myocardial infarction. Possible pro-atherogenic mechanisms of these inhibitors have been suggested, including an imbalance in prostanoid production leaving the pro-aggregatory prostaglandins unopposed, but the precise mechanisms involved have not been elucidated. We explored the possibility that downregulation of proteins involved in reverse cholesterol transport away from atheromatous plaques contributes to increased atherogenesis associated with COX inhibition. The reverse cholesterol transport proteins cholesterol 27-hydroxylase and ATP-binding cassette transporter A1 (ABCA1) export cholesterol from macrophages. When mechanisms to process lipid load are inadequate, uncontrolled cholesterol deposition in macrophages transforms them into foam cells, a key element of atheromatous plaques. We showed that in cultured THP-1 human monocytes/macrophages, inhibition of COX-1, COX-2, or both reduced expression of 27-hydroxylase and ABCA1 message (real-time reverse transcription-polymerase chain reaction) and protein (immunoblot). The selective COX-2 inhibitor *N*-(2-cyclohexyloxy-4-nitrophenyl)methanesulfonamide (NS398) significantly reduced 27-hydroxylase and ABCA1 message (to 62.4% ± 2.2% and 71.1% ± 3.9% of control, respectively). Incubation with prostaglandin (PG) E_2 _or PGD_2 _reversed reductions in both of these cholesterol transport proteins induced by NS398. Cholesterol-loaded THP-1 macrophages showed significantly increased foam cell transformation in the presence of NS398 versus control (42.7% ± 6.6% versus 20.1% ± 3.4%, *p *= 0.04) as determined by oil red O staining. Pharmacological inhibition of COX in monocytes is involved in downregulation of two proteins that mediate cholesterol efflux: cholesterol 27-hydroxylase and ABCA1. Because these proteins are anti-atherogenic, their downregulation may contribute to increased incidence of cardiac events in patients treated with COX inhibitors. Reversal of inhibitory effects on 27-hydroxylase and ABCA1 expression by PGD_2 _and PGE_2 _suggests involvement of their respective signaling pathways. NS398-treated THP-1 macrophages show greater vulnerability to form foam cells. Increased cardiovascular risk with COX inhibition may be ascribed at least in part to altered cholesterol metabolism.

## Introduction

Both non-selective cyclooxygenase (COX) inhibitors and selective inhibitors of COX-2 are effective anti-inflammatory and analgesic drugs that exert their action by preventing the formation of prostanoids [1-3]. Based on findings from the APPROVe (Adenomatous Polyp Prevention on Vioxx) trial, the COX-2 inhibitor rofecoxib was withdrawn from the market due to a significant increase in the incidence of cardiovascular events in subjects treated with rofecoxib compared with placebo (relative risk 1.92, 95% confidence interval [CI] 1.19 to 3.11) [4]. Subsequently, the COX-2 inhibitor Bextra (valdecoxib) was withdrawn from the market because it too was found to significantly increase the risk of myocardial infarction (MI) and stroke.

Although COX-2 inhibitors elevate heart attack and stroke incidence up to three-fold, the mechanisms by which selective inhibitors of COX-2 might predispose individuals to heart disease and stroke are incompletely understood. It has been hypothesized that selective COX-2 inhibition upsets the thrombotic equilibrium and creates an imbalance between anti-thrombotic and pro-thrombotic factors by blocking endothelium-derived prostaglandin (PG) I_2 _while sparing platelet-derived thromboxane [5,6]. A meta-analysis of randomized trials demonstrated a dose-dependent increase in cardiovascular events with COX-2 inhibitors which begins early in treatment [7]. High-dose regimens of some traditional non-selective COX inhibitors (non-steroidal anti-inflammatory drugs [NSAIDs]) such as diclofenac and ibuprofen are under scrutiny and have been associated with increased risk of MI [8].

The promotion of platelet aggregation by COX-2 inhibition is the predominant theory to explain increased cardiovascular events [5,6]. However, abnormal cholesterol deposition in the coronary arteries is a strong component of atherosclerosis [9]. The biologic mechanisms of COX inhibition with respect to cholesterol metabolism have not been evaluated. We previously reported that immune reactants, including interferon-gamma (IFN-γ) and immune complex-C1q complexes, diminish expression of both cholesterol 27-hydroxylase, an anti-atherogenic enzyme, and ATP-binding cassette transporter A1 (ABCA1), a protein that controls a cellular pathway for secretion of cholesterol for transport to the liver, in cells relevant to atherogenesis [10,11]. We therefore investigated the effect of COX inhibition on cholesterol transport proteins in human monocytes/macrophages. Our data demonstrate that pharmacological inhibition of COX reduces expression of the cholesterol-metabolizing enzyme cholesterol 27-hydroxylase and the cholesterol transport protein ABCA1. Because these proteins are usually atheroprotective [11,12], their downregulation may contribute to a propensity toward atherogenesis as a result of COX inhibition.

## Materials and methods

### Reagents

Oil red O was purchased from Sigma-Aldrich (St. Louis, MO, USA). Trizol reagent was purchased from Invitrogen Corporation (Carlsbad, CA, USA). All reagents for reverse transcription and quantitative real-time polymerase chain reaction (QRT-PCR) were purchased from Applied Biosystems (Foster City, CA, USA). Recombinant human IFN-γ was purchased from R&D Systems, Inc. (Minneapolis, MN, USA). Acetylated low-density lipoprotein (LDL) was purchased from Intracel Resources, LLC (Frederick, MD, USA). Anti-cholesterol 27-hydroxylase antibody is an affinity-purified rabbit polyclonal anti-peptide antibody raised against residues 15 to 28 of the cholesterol 27-hydoxylase protein [13]. Anti-human ABCA1 antibody was purchased from Santa Cruz Biotechnology, Inc (Santa Cruz, CA, USA). *N*-(2-cyclohexyloxy-4-nitrophenyl)methanesulfonamide (NS398) was purchased from Sigma/RBI (Natick, MA, USA). Indomethacin was obtained from Sigma-Aldrich. Prostaglandins, 5-(4-Chlorophenyl)-1-(4-methoxyphenyl)-3-trifluoromethylpyrazol (SC560), and thromboxane A_2 _(TXA_2_) were obtained from Cayman Chemical Company (Ann Arbor, MI, USA).

### Cell culture

THP-1 monocytes (American Type Culture Collection, Manassas, VA, USA) were grown at 37°C in a 5% CO_2 _atmosphere to a density of 10^6 ^cells per milliliter. Growth medium for THP-1 cells was RPMI 1640 (GIBCO BRL, now part of Invitrogen Corporation) supplemented with 10% fetal bovine serum (Invitrogen Corporation), 50 units per milliliter penicillin, and 50 units per milliliter streptomycin. THP-1 cells then were subjected to the experimental conditions described or were differentiated into adherent macrophages (phorbol dibutyrate, 0.3 μM, 48 hours).

### Experimental conditions

When THP-1 monocytes reached 10^6 ^cells per milliliter, media was aspirated and cells were rinsed twice with Dulbecco's phosphate-buffered saline (PBS) without calcium and magnesium. The cells were then incubated (18 to 24 hours, 37°C, 5% CO_2_) in six-well plates under the following conditions: (a) RPMI control, (b) RPMI containing NS398 (10 to 100 μM), (c) RPMI containing indomethacin (0.5, 5, and 50 μM), (d) RPMI containing SC560 (0.001 to 0.1 μM), and (e) RPMI containing NS398 and TXA_2 _(3 μM). Immediately after the incubation period, the cells were collected and centrifuged at 1,500 rpm at room temperature, media was aspirated, and cell protein and RNA were isolated.

THP-1 monocytes (10^6 ^cells per milliliter) were converted to macrophages (phorbol dibutyrate, 0.3 μM, 48 hours) and then incubated with NS398 (50 μM) for 24 hours followed by the addition of PGD_2_, PGE_2_, or TXA_2 _for a further 24 hours. Immediately after the incubation period, total RNA was isolated. Concentrations of inhibitors used were in the range of prior *in vivo *and *in vitro *studies [14-16].

### Trypan blue exclusion assay

Cell viability was determined using the vital dye trypan blue, which is excluded by living cells but accumulates in dead cells. THP-1 cells treated as indicated were stained with 0.4% trypan blue solution (Sigma-Aldrich). Cell death was expressed as the percentage of trypan blue-stained cells. Assays were performed at least three times.

### RNA isolation

RNA was isolated using 1 ml of Trizol reagent per 10^6 ^cells and dissolved in nuclease-free water. The quantity of total RNA from each condition was measured by absorption at 260- and 280-nm wavelengths using quartz cuvettes by ultraviolet spectrophotometry (Hitachi U2010 spectrophotometer; Hitachi, Ltd., Tokyo, Japan).

### Analysis of 27-hydroxylase and ABCA1 message by QRT-PCR

All reverse transcription reactions were carried out in an Eppendorf Mastercycler^®^-personal (Eppendorf, Hamburg, Germany) as previously described [10]. QRT-PCR was performed after reverse transcription of 5 μg of total RNA into cDNA. QRT-PCR analysis was performed using the SYBR Green PCR Reagents Kit (Applied Biosystems) with a Stratagene MX3005P QPCR System (Stratagene, La Jolla, CA, USA) according to the manufacturers' instructions. RNA was isolated from cells grown on a 60 × 15 mm dish as described previously [10,17] and quantified on a spectrophotometer at 260 nm. cDNA was copied from 5 μg of total RNA using MMLV (Moloney murine leukemia virus) reverse transcriptase primed with oligo dT. cDNA was amplified with specific primers (48 pmol/reaction) for ABCA1 (forward primer 5'-GAAGTACATCAGAACATGGGC-3' and reverse primer 5'-GATCAAAGCCATGGCTGTAG-3' with 234-base pair amplified fragment) and 27-hydroxylase. The cholesterol 27-hydroxylase-specific primers span a 311-base pair sequence encompassing nucleotides 491 to 802 of the human cholesterol 27-hydroxylase cDNA [17].

PCR was performed using techniques standardized in our laboratory. Each PCR reaction contained 2.5 μl of the 10× fluorescent green buffer, 3 μl of 25 mM MgCl_2_, 2 μl of dNTP mix (2,500 μM dCTP, 2,500 μM dGTP, 2,500 μM dATP, and 5,000 μM dUTP), 0.15 μl of polymerase (5 U/μl; AmpliTaq Gold; Applied Biosystems), 0.25 μl uracil-N-glycosylase (1 U/μl; AmpErase; Applied Biosystems), 0.5 μl of the forward and reverse primers (10-μM concentration), 4 μl of cDNA, and water to a final volume of 25 μl. The thermal cycling parameters were as follows: 5 minutes at 95°C to activate the polymerase (AmpliTaq Gold; Applied Biosystems) followed by 45 cycles of 30 seconds at 95°C, 45 seconds at 58°C, and 45 seconds at 72°C. Each reaction was done in triplicate. The amounts of PCR products were estimated using software provided by the manufacturer (Stratagene). After completion of PCR cycles, the reactions were heat-denatured over a 35°C temperature gradient from 60°C to 95°C. To correct for differences in cDNA load among samples, the target PCRs were normalized to a reference PCR involving the endogenous housekeeping genes GAPDH (glyceraldehyde-3-phosphate dehydrogenase) and β-actin. Non-template controls were included for each primer pair to check for significant levels of any contaminants. Fluorescence emission spectra were monitored and analyzed. PCR products were measured by the threshold cycle (C_T_) values, at which specific fluorescence becomes detectable. The C_T _was used for kinetic analysis and was proportional to the initial number of target quantity copies in the sample. A melting-curve analysis was performed to assess the specificity of the amplified PCR products. The quantity of the samples was calculated after the C_T _values of the serial dilutions were compared with a control. QRT-PCR standards were prepared by making 1:10 serial dilutions of a purified PCR product.

### Protein extraction and Western blot analysis

Total cell lysates were prepared for Western immunoblotting using radioimmunoprecipitation assay (RIPA) lysis buffer (98% PBS, 1% Igepal [polyoxyethylene nonylphenol] CA-630, 0.5% sodium deoxycholate, 0.1% SDS). One hundred microliters of RIPA lysis buffer and 10 μl of protease inhibitor cocktail (Sigma-Aldrich) were added to the cell pellet from each condition and incubated on ice for 35 minutes with vortexing every 5 minutes. Supernatants were collected after centrifuging at 10,000 *g *at 4°C for 10 minutes using an Eppendorf 5415C centrifuge. The quantity of protein in each supernatant was measured by absorption at 560 nm using a Hitachi U2010 spectrophotometer (Hitachi, Ltd.).

Total cell lysate was used for Western blots. Protein samples (20 μg/lane) were boiled for 5 minutes, loaded onto a 10% polyacrylamide gel, electrophoresed for 1.5 hours at 100 V, and transferred to a nitrocellulose membrane in a semi-dry transblot apparatus for 1 hour at 100 V. The nitrocellulose membrane was blocked for 4 hours at 4°C in blocking solution (3% non-fat dry milk dissolved in 1 × Tween20-tris-buffered saline [TTBS]) and then immersed in a 1:300 dilution of primary antibody (18.7 μg/ml) in blocking solution overnight at 4°C. The primary antibody is an affinity-purified rabbit polyclonal anti-peptide antibody raised against residues 15 to 28 of the cholesterol 27-hydroxylase protein [11]. The following day, the membrane was washed five times in TTBS for 5 minutes per wash and then incubated at room temperature in a 1:3,000 dilution of enhanced chemiluminescence (ECL) donkey anti-rabbit immunoglobulin G (IgG) horseradish peroxidase-linked species-specific whole antibody (product code NA934; Amersham Biosciences, now part of GE Healthcare, Little Chalfont, Buckinghamshire, UK). The five washes in TTBS were repeated, and then the immunoreactive protein was detected using ECL Western blotting detection reagent (catalog number RPN2106; GE Healthcare) and film development in SRX-101A (Konica Minolta Holdings, Inc., Tokyo, Japan).

As control, on the same transferred membrane, β-actin was detected using mouse anti-β-actin (diluted in 1:1,000) (product code ab6276; Abcam, Cambridge, UK) and ECL sheep anti-mouse-IgG horseradish peroxidase-linked species-specific whole antibody (diluted in 1:2,000) (product code NA931; GE Healthcare) and all other similar steps as above.

For ABCA1 detection, macrophage cell lysates were electrophoresed for 1.5 hours at 100 V (10% polyacrylamide gel) and then transferred to a nitrocellulose membrane. The membrane was blocked for 4 hours at 4°C in blocking solution and then incubated overnight at 4°C in a 1:200 dilution of rabbit anti-ABCA1 antibody (Santa Cruz Biotechnology, Inc.). The following day, the membrane was washed five times in TTBS for 5 minutes per wash and then incubated at room temperature in a 1:5,000 dilution of ECL donkey anti-rabbit IgG horseradish peroxidase-linked species-specific whole antibody. Development proceeded as described above for the 27-hydroxylase antibody.

### Statistical analysis of experimental data

Statistical analysis was performed using SigmaStat version 2.03 (SPSS Inc., Chicago, IL, USA). Data was analyzed using the Kruskal-Wallis one-way analysis of variance on ranks. Pairwise multiple comparison was made with the Holm-Sidak method.

### Foam cell formation and staining

THP-1 human monocytes (10^6 ^cells per milliliter) in 12-well plates were treated with phorbol dibutyrate (0.3 μM) (Sigma-Aldrich) for 48 hours at 37°C to facilitate differentiation into macrophages. The differentiated macrophages were washed three times with PBS and then incubated alone or in the presence of 10 or 50 μM NS398 (37°C, 5% CO_2_, 18 hours). Cells were cholesterol-loaded with acetylated LDL (50 μg/ml) and further incubated in RPMI (37°C, 5% CO_2_) for 48 hours under the following conditions: (a) control, (b) PGD_2 _(14 μM), (c) PGE_2 _(0.1 μM), and (d) PGI_2 _(0.1 μM). Studies were performed in triplicate.

Immediately after incubation, media was aspirated and cells were fixed in the same 12-well plates used for incubation, with 4% paraformaldehyde in water, for 2 to 4 minutes. Cells were stained with 0.2% oil red O in methanol for 1 to 3 minutes. Cells were observed via light microscope (Axiovert 25-Zeiss; Carl Zeiss, Jena, Germany) with ×100 magnification and then photographed using a Kodak DC 290 Zoom Digital Camera (Eastman Kodak, Rochester, NY, USA). The numbers of foam cells formed in each condition were calculated manually and presented as the percentage of foam cell formation.

## Results

### COX-2 inhibition decreases 27-hydroxylase and ABCA1 in THP-1 monocytes

**Figure 1:**
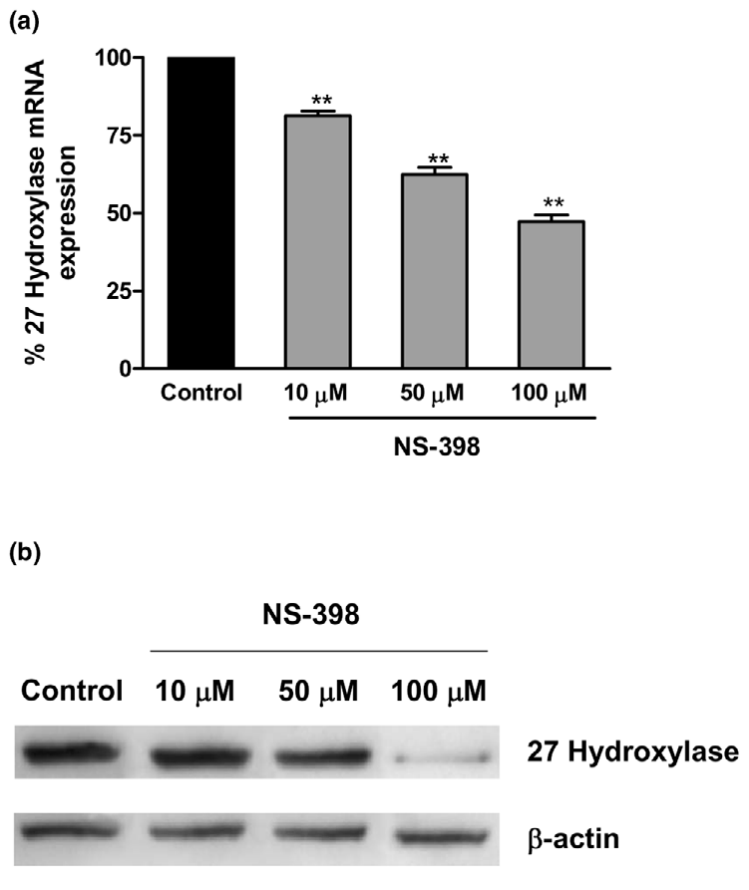
Detection and quantitation of cholesterol 27-hydroxylase in THP-1 cells exposed to NS398. **(a) **Dose-dependent decrease in 27-hydroxylase mRNA expression in THP-1 monocytes treated with the COX-2 inhibitor NS398. Cultured THP-1 monocytic cells were untreated or exposed to NS398 for 18 hours. After isolation of total RNA, the RNA was reverse-transcribed and the cDNA amplified by quantitative real-time polymerase chain reaction as described. Signals obtained from the amplification of GAPDH message were used as internal controls. **(b) **Dose-dependent decrease in 27-hydroxylase protein expression in THP-1 monocytes treated with the COX-2 inhibitor NS398. Cultured THP-1 monocytic cells were untreated or exposed to NS398 for 18 hours. Total cell protein was isolated and 27-hydroxylase detected with specific rabbit polyclonal anti-human 27-hydroxylase antibody. Western blotting was performed with an anti-β-actin antibody to confirm equal protein loading. At 100 mM NS398 concentration, cell death was statistically significant (14.8% ± 6.3%). COX, cyclooxygenase; GAPDH, glyceraldehyde-3-phosphate dehydrogenase; NS398, *N*-(2-cyclohexyloxy-4-nitrophenyl)methanesulfonamide. ** *p *< 0.01.

Exposure to NS398 markedly reduced cholesterol 27-hydroxylase message expression by THP-1 monocytes (50 μM, 62.4% ± 2.2% of control, *n *= 3, *p *< 0.001) (Figure 1a). Western blotting with a rabbit polyclonal anti-27-hydroxylase antibody [11] showed a concomitant decrease in 27-hydroxylase protein in THP-1 monocytes exposed to NS398 (Figure 1b).

**Figure 2:**
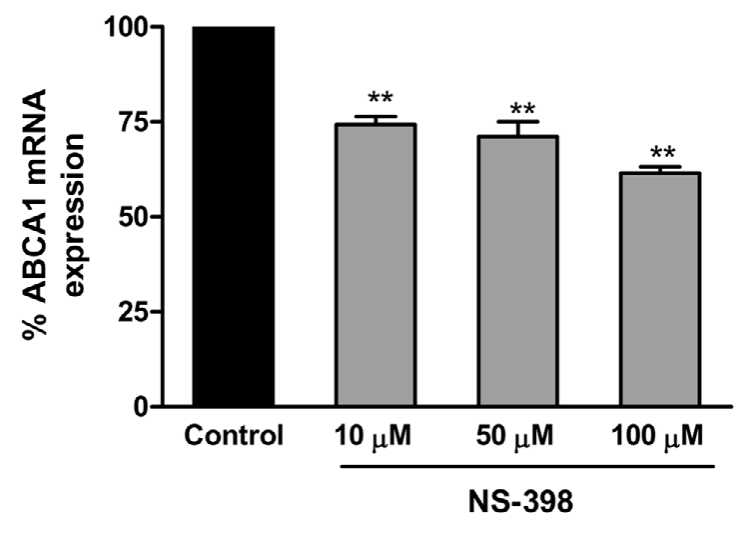
Quantitation of ABCA1 message in THP-1 cells exposed to NS398. Dose-dependent decrease in ABCA1 mRNA expression in THP-1 monocytes treated with the COX-2 inhibitor NS398. Cultured THP-1 monocytic cells were untreated or exposed to NS398 for 18 hours. After isolation of total RNA, the RNA was reverse-transcribed and the cDNA amplified by quantitative real-time polymerase chain reaction as described. Signals obtained from the amplification of GAPDH message were used as internal controls. At 100 mM NS398 concentration, cell death was statistically significant (14.8% ± 6.3%). ABCA1, ATP-binding cassette transporter A1; COX, cyclooxygenase; GAPDH, glyceraldehyde-3-phosphate dehydrogenase; NS398, *N*-(2-cyclohexyloxy-4-nitrophenyl)methanesulfonamide. ** *p *< 0.01.

ABCA1 is a key membrane-associated protein involved in reverse cholesterol transport. Similar to 27-hydroxylase, ABCA1 message was reduced after NS398 exposure to approximately 70% of control (50 μM, 71.1% ± 3.9% of control, *n *= 3, *p *< 0.01) (Figure 2).

NS398-induced reductions in 27-hydroxylase and ABCA1 message were observed as early as 3 hours and maintained through 72 hours (data not shown). These results suggest that modulation of cellular mechanisms intricately involved in cholesterol flux may be responsible for the atherogenicity of COX inhibitors. We therefore examined the effect of non-selective and selective COX-1 inhibitors on 27-hydroxylase and ABCA1 expression.

### Non-selective COX inhibition reduces 27-hydroxylase and ABCA1 expression

**Figure 3:**
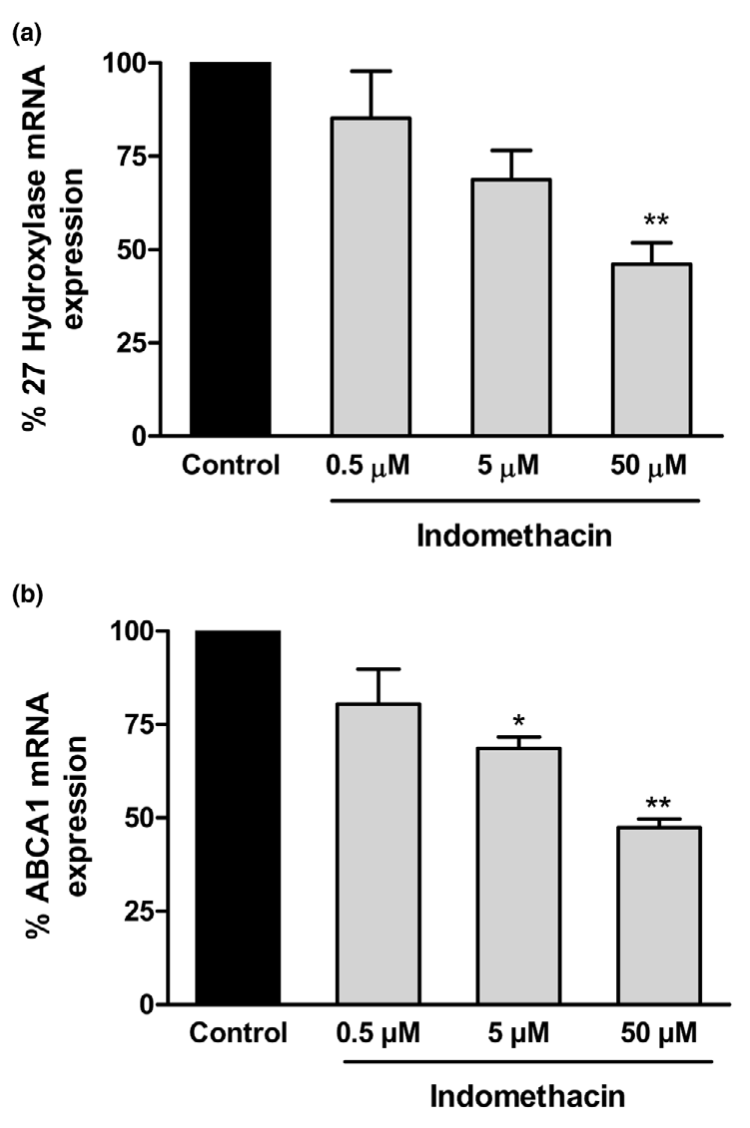
QRT-PCR for 27-hydroxylase and ABCA1 message in indomethacin-treated THP-1 cells. **(a) **27-Hydroxylase mRNA expression is decreased by the non-specific COX inhibitor indomethacin in a dose-dependent fashion in THP-1 monocytes. Cultured THP-1 monocytic cells were untreated or exposed to increasing doses of indomethacin for 18 hours. After isolation of total RNA, the RNA was reverse-transcribed and the cDNA amplified by QRT-PCR as described. Signals obtained from the amplification of GAPDH message were used as internal controls. **(b) **ABCA1 mRNA expression is decreased by the non-specific COX inhibitor indomethacin in a dose-dependent fashion in THP-1 monocytes. Cultured THP-1 monocytic cells were untreated or exposed to increasing doses of indomethacin for 18 hours. After isolation of total RNA, the RNA was reverse-transcribed and the cDNA amplified by QRT-PCR as described. Signals obtained from the amplification of GAPDH message were used as internal controls. At 50 mM indomethacin concentration, cell death was statistically significant (16.8% ± 1.0%). ABCA1, ATP-binding cassette transporter A1; COX, cyclooxygenase; GAPDH, glyceraldehyde-3-phosphate dehydrogenase; QRT-PCR, quantitative real-time polymerase chain reaction. * *p *< 0.05, ** *p *< 0.01.

The non-selective COX inhibitor indomethacin also produced a significant reduction in cholesterol 27-hydroxylase and ABCA1 mRNA expression (50 μM, 46.0% ± 5.9% of control, *n *= 3, *p *< 0.01 for 27-hydroxylase; 50 μM, 47.5% ± 2.2% of control, *n *= 3, *p *< 0.001 for ABCA1) (Figure 3).

### COX-1 inhibition downregulates 27-hydroxylase

**Figure 4:**
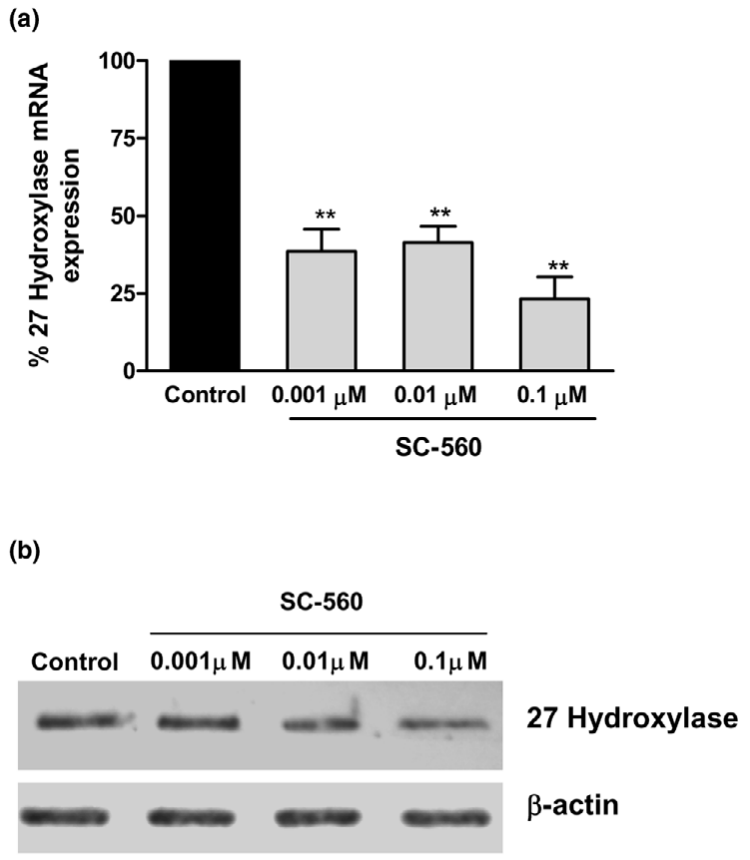
Detection and quantitation of cholesterol 27-hydroxylase in THP-1 cells exposed to SC560. **(a) **27-Hydroxylase mRNA expression in THP-1 monocytes is decreased by the specific COX-1 inhibitor SC560. Cultured THP-1 monocytic cells were untreated or exposed to increasing doses of SC560 for 24 hours. After isolation of total RNA, the RNA was reverse-transcribed and the cDNA amplified by quantitative real-time polymerase chain reaction as described. Signals obtained from the amplification of GAPDH message were used as internal controls. **(b) **Decrease in 27-hydroxylase protein expression in THP-1 monocytes treated with the COX-1 inhibitor SC560. Cultured THP-1 human monocytes were untreated or exposed to SC560 for 24 hours. Total cell protein was isolated and 27-hydroxylase detected with specific rabbit polyclonal anti-human 27-hydroxylase antibody. Western blotting was performed with an anti-β-actin antibody to confirm equal protein loading. COX, cyclooxygenase; GAPDH, glyceraldehyde-3-phosphate dehydrogenase; SC560, 5-(4-Chlorophenyl)-1-(4-methoxyphenyl)-3-trifluoromethylpyrazol. ** *p *< 0.01.

The COX-1 inhibitor SC560 reduces 27-hydroxylase mRNA expression in THP-1 monocytes (at 0.1 μM, 23.3% ± 7.0% of control, *n *= 3, *p *< 0.001) (Figure 4a). These results are confirmed at the protein level by Western blotting (Figure 4b).

### COX-2 inhibitor-mediated downregulation of 27-hydroxylase and ABCA1 mRNA is reversed by prostaglandins PGE_1_, PGE_2_, and PGD_2_

**Figure 5:**
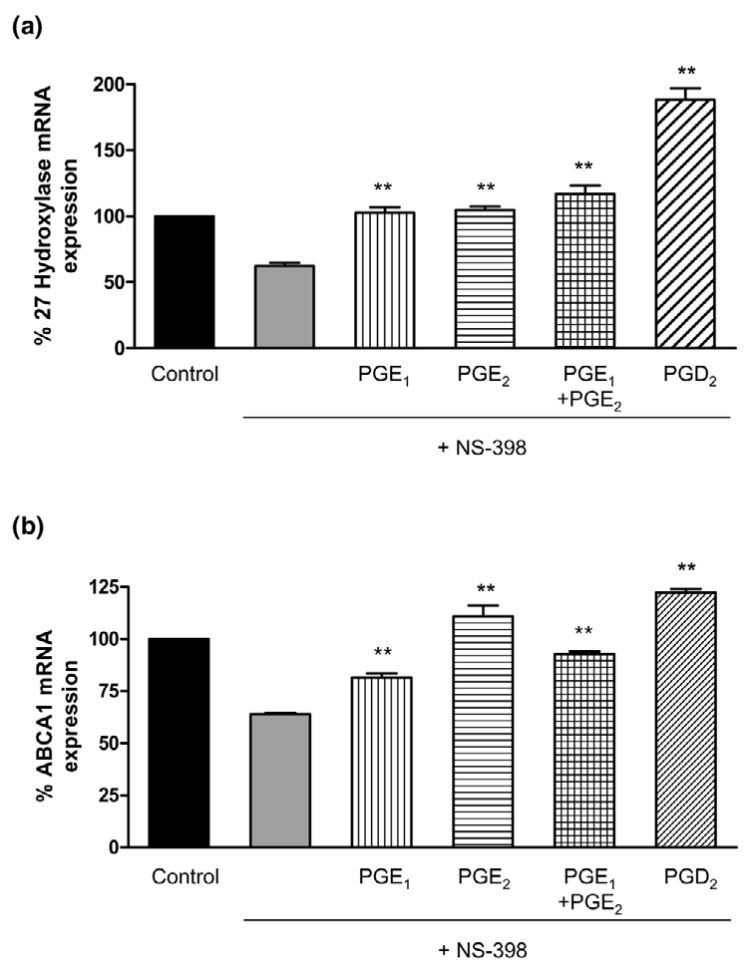
QRT-PCR for 27-hydroxylase and ABCA1 message in NS398-treated THP-1 cells exposed to prostaglandins. **(a) **27-Hydroxylase message is decreased by the COX-2 inhibitor NS398 and this decrease is reversed by prostaglandins E_1_, E_2_, and D_2_. THP-1 human monocytes were exposed to the following conditions represented by the six bars (from left to right): (1) RPMI 1640, (2) NS398 (50 μM), (3) PGE_1 _(0.1 μM) + NS398 (50 μM), (4) PGE_2 _(0.1 μM) + NS398 (50 μM), (5) PGE_1 _(0.1 μM) + PGE_2 _(0.1 μM) + NS398 (50 μM), and (6) PGD_2 _(14 μM) + NS398 (50 μM) (all 18-hour exposures). Cells were extracted for total RNA and were evaluated for 27-hydroxylase mRNA expression by QRT-PCR. Signals obtained from the amplification of GAPDH message were used as internal controls. **(b) **ABCA1 message is decreased by the COX-2 inhibitor NS398 and this decrease is reversed by prostaglandins E_1_, E_2_, and D_2_. THP-1 human monocytes were exposed to the following conditions represented by the six bars (from left to right): (1) RPMI 1640, (2) NS398 (50 μM), (3) PGE_1 _(0.1 μM) + NS398 (50 μM), (4) PGE_2 _(0.1 μM) + NS398 (50 μM), (5) PGE_1 _(0.1 μM) + PGE_2 _(0.1 μM) + NS398 (50 μM), and (6) PGD_2 _(14 μM) + NS398 (50 μM) (all 18-hour exposures). Cells were extracted for total RNA and were evaluated for ABCA1 mRNA expression by QRT-PCR. Signals obtained from the amplification of GAPDH message were used as internal controls. **p < 0.01 compared to NS398 (*n *= 5). ABCA1, ATP-binding cassette transporter A1; COX, cyclooxygenase; GAPDH, glyceraldehyde-3-phosphate dehydrogenase; NS398, *N*-(2-cyclohexyloxy-4-nitrophenyl)methanesulfonamide; PG, prostaglandin; QRT-PCR, quantitative real-time polymerase chain reaction.

Effects of the specific COX-2 inhibitor NS398 on 27-hydroxylase and ABCA1 level in THP-1 monocytes were reversed by PGE_1_, PGE_2_, and PGD_2 _prostaglandin products of COX-2 (Figure 5).

### TXA_2 _failed to reverse COX-2 inhibitor-mediated downregulation of 27-hydroxylase and ABCA1

**Figure 6:**
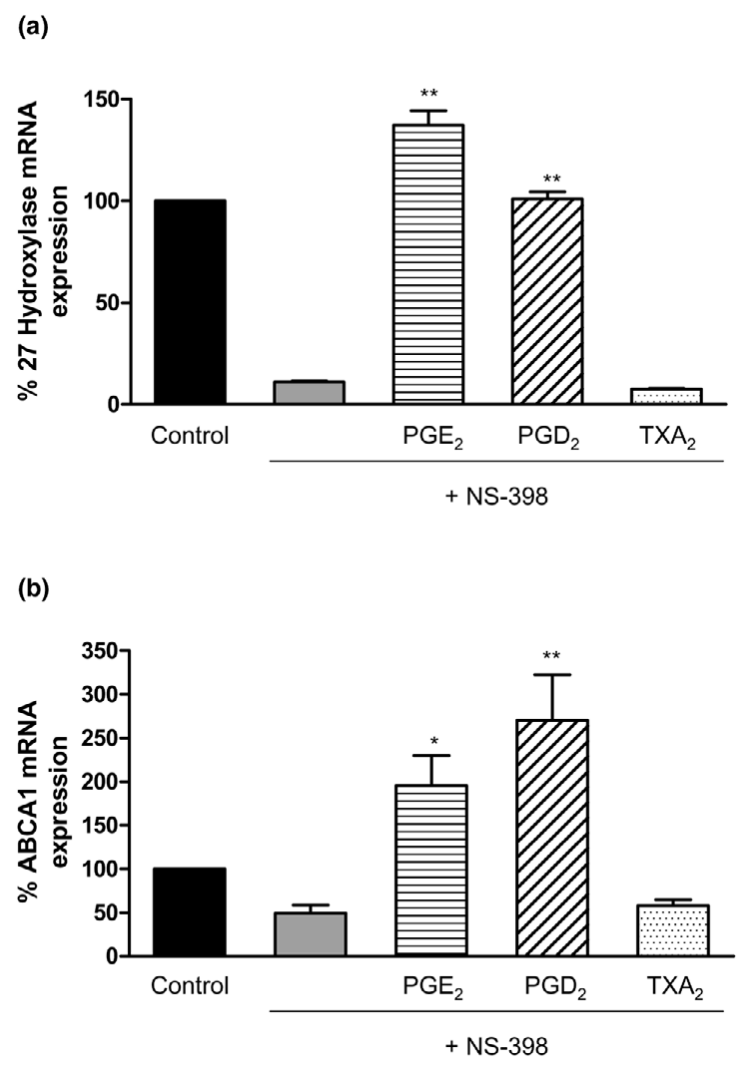
QRT-PCR for 27-hydroxylase and ABCA1 message in NS398-treated THP-1 macrophages exposed to prostaglandins or TXA_2_. **(a) **27-Hydroxylase message in THP-1 macrophages is decreased by the COX-2 inhibitor NS398 and this decrease is reversed by prostaglandins E_2 _and D_2_, but not TXA_2_. THP-1 human macrophages were exposed to the following conditions represented by the five bars (from left to right): (1) RPMI 1640, (2) NS398 (50 μM), (3) PGE_2 _(0.1 μM) + NS398 (50 μM), (4) PGD_2 _(14 μM) + NS398 (50 μM), and (5) TXA_2 _(3 μM) + NS398 (50 μM) (24-hour exposures to NS398 alone followed by addition of indicated PG or TXA_2 _for a further 24 hours). Cells were extracted for total RNA and were evaluated for 27-hydroxylase mRNA expression by QRT-PCR. Signals obtained from the amplification of GAPDH message were used as internal controls. **(b) **ABCA1 message is decreased by the COX-2 inhibitor NS398 in THP-1 macrophages and this decrease is reversed by prostaglandins E_2 _and D_2_, but not TXA_2_. THP-1 human macrophages were exposed to the following conditions represented by the five bars (from left to right): (1) RPMI 1640, (2) NS398 (50 μM), (3) PGE_2 _(0.1 μM) + NS398 (50 μM), (4) PGD_2 _(14 μM) + NS398 (50 μM), and (5) TXA_2 _(3 μM) + NS398 (50 μM) (24-hour exposures to NS398 alone followed by addition of indicated PG or TXA_2 _for a further 24 hours). Cells were extracted for total RNA and were evaluated for ABCA1 mRNA expression by QRT-PCR. Signals obtained from the amplification of GAPDH message were used as internal controls. *p < 0.05, **p < 0.01 compared to NS398 (*n *= 3). ABCA1, ATP-binding cassette transporter A1; COX, cyclooxygenase; GAPDH, glyceraldehyde-3-phosphate dehydrogenase; NS398, *N*-(2-cyclohexyloxy-4-nitrophenyl)methanesulfonamide; PG, prostaglandin; QRT-PCR, quantitative real-time polymerase chain reaction; TXA_2_, thromboxane A_2_.

Reversal of NS398-induced downregulation of 27-hydroxylase and ABCA1 message by PGE_2 _and PGD_2 _was also observed in THP-1 macrophages (Figure 6). However, TXA_2 _failed to reverse the effect of NS398 on either 27-hydroxylase or ABCA1 message. Further verifying the ineffectiveness of TXA_2_, NS398-induced diminution of 27-hydroxylase protein level was not restored by TXA_2 _(Western blot not shown).

### COX-2 inhibition decreases ABCA1 protein and increases foam cell formation in THP-1 macrophages

**Figure 7:**
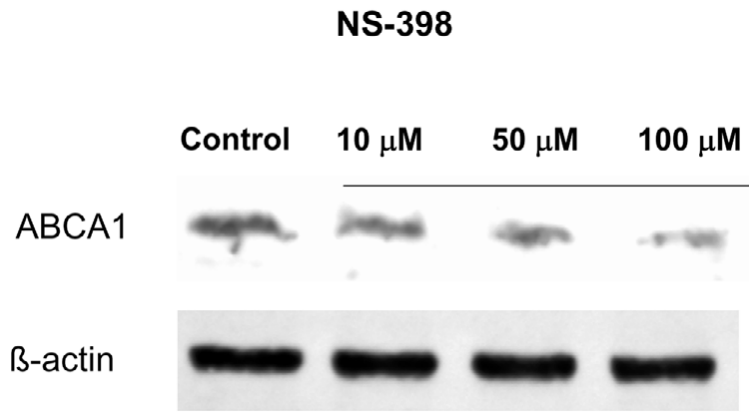
Decrease in ABCA1 protein in THP-1 macrophages exposed to NS398. Cultured THP-1 human macrophages were untreated or exposed to increasing concentrations of NS398 for 18 hours. Total cell protein was isolated and ABCA1 detected with specific rabbit polyclonal anti-ABCA1 antibody. Western blotting was performed with an anti-β-actin antibody to confirm equal protein loading. ABCA1, ATP-binding cassette transporter A1; NS398, *N*-(2-cyclohexyloxy-4-nitrophenyl)methanesulfonamide.

**Figure 8:**
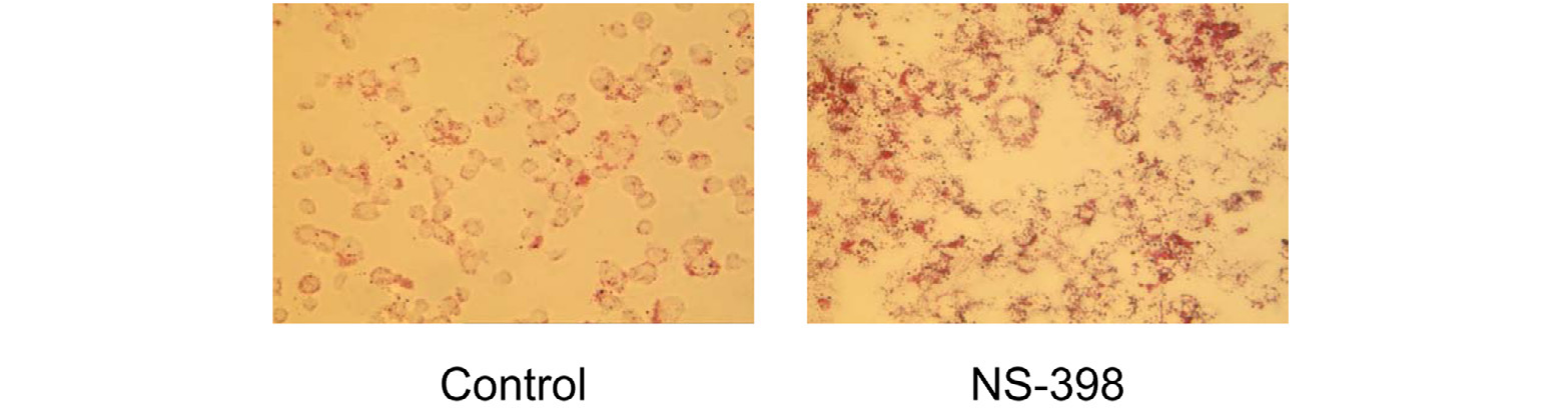
NS398 increases foam cell formation in THP-1 macrophages. THP-1 differentiated macrophages were treated with acetylated low-density lipoprotein (50 μg/ml, 48 hours) and further incubated alone or with the addition of NS398 (10 μM, 18 hours). Representative photomicrographs of oil red O staining to detect foam cells. NS398, *N*-(2-cyclohexyloxy-4-nitrophenyl)methanesulfonamide.

THP-1 macrophages exposed to the selective COX-2 inhibitor NS398 showed a dose-dependent decrease in ABCA1 protein level (Figure 7). Under conditions of cholesterol loading with acetylated LDL, THP-1 macrophages treated with NS398 exhibited greater propensity to form lipid-laden foam cells as compared to untreated cells. THP-1 macrophages showed a significant increase in foam cell transformation in the presence of NS398 compared to control (78.9% ± 4.4% at 10 μM NS398 versus 52.1% ± 5.2% untreated, *p *< 0.05, and 89.0% ± 2.3% at 50 μM NS398 versus 39.6% ± 5.7% untreated, *p *< 0.001; *n *= 3 for each) (Figure 8).

PGD_2 _(14 μM) and PGE_2 _(0.1 μM) decreased foam cell formation in NS398 (50 μM)-treated macrophages by 34.6% ± 5.5% and 37.6% ± 6.5%, respectively (*n *= 3, *p *< 0.001). PGI_2 _(0.1 μM) did not reverse NS398-induced foam cell transformation. Selective inhibition of COX-1 with SC560 (0.001 μM) also increased foam cell transformation (87% ± 10% above control, *n *= 3, *p *< 0.001). PGD_2 _did not inhibit foam cell formation in SC560-treated THP-1 macrophages.

### Cell viability

Cell viability was assessed using the trypan blue exclusion assay. Trypan blue staining showed no difference in cell viability between control and cells treated with NS398 at 10 or 50 μM. However, NS398 at a concentration of 100 μM significantly increased cell death (2.1% ± 1.7% versus 14.8% ± 6.3%, control versus NS398, 100 μM, *n *= 4, *p *< 0.05). Trypan blue staining similarly showed no difference in cell viability between control and cells treated with indomethacin at 0.5 or 5 μM. However, indomethacin at a concentration of 50 μM significantly increased cell death (2.0% ± 0.6% versus 16.8% ± 1.0%, control versus indomethacin, 50 μM, *n *= 3, *p *< 0.001). In cell samples treated with SC560 at 0.001, 0.01, or 0.1 μM, trypan blue staining demonstrated no difference in cell viability between control and treatment groups at all concentrations (*n *= 3, *p *= not significant). Addition of prostaglandins did not affect cell viability.

## Discussion

Selective COX-2 inhibitors reduce pain, stiffness, and inflammation with efficacy equivalent to non-selective NSAIDs, but with reduced gastrotoxicity [18]. Unfortunately, adverse effects on coronary heart disease risk with prolonged use of COX-2s may offset any gastrointestinal benefit. The increased cardiovascular risk of COX-2s is attributed to a pro-thrombotic vascular environment resulting from suppression of PGI_2_, a potent vasodilator and inhibitor of platelet aggregation, without a balancing effect on TXA_2_, a platelet activator, vasoconstrictor, and smooth muscle mitogen [5,6]. Little is known of the impact of these drugs on the cholesterol transport system.

COX enzymes catalyze the rate-limiting step in the prostanoid biosynthesis pathway, converting arachidonic acid into the chemically unstable intermediate PGH_2_, from which prostaglandins and thromboxanes are derived. Atherosclerosis is associated with an increase in prostaglandin biosynthesis [19] and COX-2 may be responsible for this increase. Expression of COX-2 has also been specifically linked to vascular wall pathology. Protein extracts from healthy arteries contain constitutive COX-1 only, but atheromatous lesions contain both COX-1 and COX-2 protein [19]. COX-2 protein levels are elevated in endothelial cells, smooth muscle cells, and macrophages in human atherosclerotic lesions [20,21]. In a rabbit model of dietary cholesterol-induced cardiovascular disease, COX-2 expression was induced in atherosclerotic plaques and may play a role in altering localized synthesis of prostanoids in these lesions [22].

**Figure 9:**
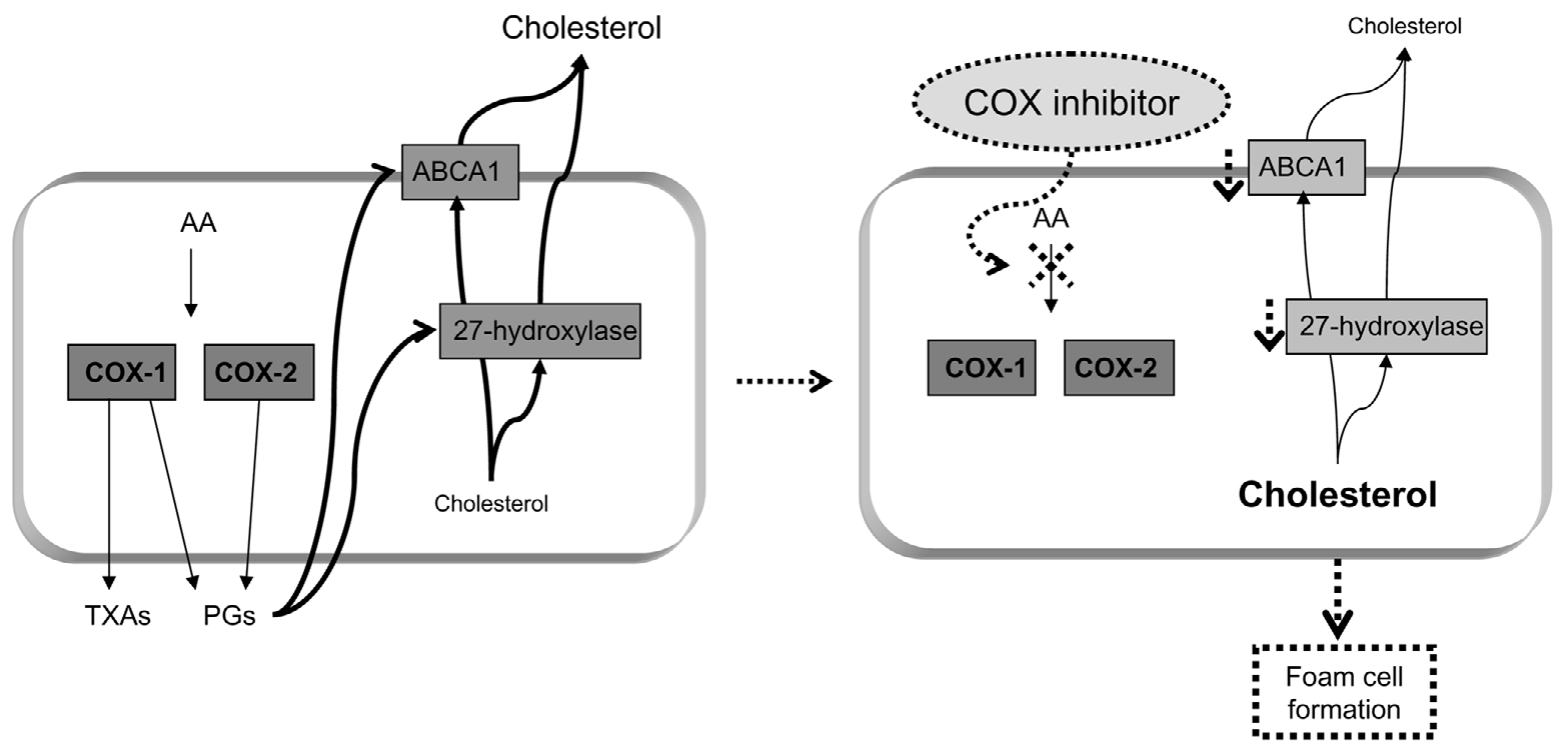
COX inhibition impairs reverse cholesterol transport. COX-1/2 inhibition downregulates 27-hydroxylase and ABCA1, thereby decreasing cholesterol efflux, in turn promoting the accumulation of cholesterol in macrophages that transform into foam cells. This effect is restored by the addition of prostaglandins. AA, arachidonic acid; ABCA1, ATP-binding cassette transporter A1; COX, cyclooxygenase; PG, prostaglandin; TXA, thromboxane A.

However, on an atherosclerosis-prone Apobec-1 and LDL receptor double-knockout murine model, Egan and colleagues [23] have shown that unlike indomethacin, urinary excretion of only PGI-M (but not other major metabolites of TXA_2_, TXB_2_, or prostacyclin) was reduced by COX-2 inhibition. Thus, effects of disturbance in the balance of thromboxanes and prostaglandins on platelet aggregability alone are insufficient to explain the heightened cardiovascular risk. Furthermore, the expression of COX-2 on platelets and the effect on overall platelet function are still matters of controversy [24]. In contrast, whereas healthy endothelial cells in culture express only COX-1, COX-2 can be readily induced under conditions of vascular injury [25-27]. In this respect, the microenvironment imposed on the vessel wall may be a more important determinant of cardiovascular risk than the influences of platelet function. Furthermore, almost complete thromboxane inhibition must be attained before *in vivo *effects on platelet activation are observed and this is unlikely to be achieved with serum levels attainable with standard doses of NSAIDs [28,29]. Disruption of the integrity of atheromatous plaque architecture adds to the vulnerability for *in situ *thrombus formation, and it has been suggested that combined inhibition of COX-2 and TXA_2 _could be detrimental to plaque stability [23,30]. Our results suggest that defective reverse cholesterol transport may be another important contributor to atheromatous plaque progression under conditions of COX-2 inhibition (Figure 9). Although the selective COX-2 inhibitor NS398 is not used in humans, the concentration achieved in pigs upon intravenous administration is 30 to 50 μM, comparable to the levels used in our studies [14].

Recently, Tuomisto and colleagues [31] employed microarray and RT-PCR to evaluate gene expression in PMA (phorbol 12-myristate 13-acetate)-stimulated THP-1 cells as a model of monocyte-macrophage differentiation that takes place during atherogenesis. In that study, lipid loading of macrophages with oxidized LDL, acetylated LDL, or native LDL induced the expression of COX-2 [31]. However, a number of studies have shown that oxidized LDL downregulates COX-2 expression [32-34]. In our studies, inhibition of COX-2 activity promoted foam cell formation, suggesting that COX-2 activity, and in particular the production of a COX-2-dependent prostanoid(s) in macrophages, may provide a defense against lipid overload. In this respect, regardless of the influence of oxidized LDL on COX-2 expression in macrophages, exogenous administration of COX inhibitors may exacerbate macrophage atherogenicity.

ABCA1 is a key regulator of cellular cholesterol and phospholipid transport. ABCA1 is an integral membrane protein that uses ATP as a source of energy for transporting lipids and other metabolites across membranes, where they are removed from cells by apolipoproteins such as apolipoprotein A-I [35,36]. Reduction in ABCA1 combined with reduction in 27-hydroxylase as a result of COX inhibition could create a microenvironment within the vessel wall where cholesterol efflux is compromised. COX inhibition may affect reverse cholesterol transport, demonstrating a possible mechanism by which COX inhibitors may cause early atheromatous lesions that lead to increased cardiovascular events. Modulation of this pro-atherogenic effect without diminution of clinical pain-relieving and anti-inflammatory efficacy may be possible and could lead to the development of new cardiovascular-sparing coxib drugs.

Although the withdrawal of rofecoxib has spawned an interest in the cardiovascular effects of COX-2 inhibition, it is of note that this observed heightened risk is not exclusive to the more selective COX-2 inhibitors but can also be observed with traditional NSAIDs [37-43]. Although naproxen was once thought to confer a protective influence on the development of cardiovascular disease, recent studies have suggested that there is in fact no benefit [44]. In a meta-analysis encompassing six studies, indomethacin was found to increase cardiovascular risk (relative risk 1.30, 95% CI 1.07 to 1.60) [7]. Interestingly, the effect of indomethacin on reverse cholesterol transport proteins in our cell culture system occurred at concentrations within the range reached with human dosing in which peak plasma levels are approximately 5 μM [15]. We have shown that the selective COX-1 inhibitor, SC560, can downregulate 27-hydroxylase expression and thereby potentially accelerate atheromatous plaque formation. This may contribute in part to the increased cardiovascular risk observed with traditional NSAIDs, although *in vitro *effects of this inhibition remain to be characterized.

## Conclusion

To our knowledge, this is the first study that describes the effects of COX inhibition on reverse cholesterol transport proteins. Our results suggest that the cardiovascular hazard observed with COX inhibitors may result not only from enhanced platelet aggregation, but also from interference with cholesterol outflow. In a rabbit model, arterial wall cholesterol content was highly correlated with severity of thrombus formation and was an independent predictor of thrombosis [41]. Further studies are necessary to determine whether the pro-thrombotic and pro-atherogenic effects of COX inhibition work in concert and to evaluate *in vivo *cholesterol metabolic changes in the presence of COX inhibition.

## Abbreviations

ABCA1 = ATP-binding cassette transporter A1; CI = confidence interval; COX = cyclooxygenase; C_T _= threshold cycle; ECL = enhanced chemiluminescence; IFN-γ = interferon-gamma; IgG = immunoglobulin G; LDL = low-density lipoprotein; MI = myocardial infarction; NSAID = non-steroidal anti-inflammatory drug; NS398 = *N*-(2-cyclohexyloxy-4-nitrophenyl)methanesulfonamide; PBS = phosphate-buffered saline; PG = prostaglandin; QRT-PCR = quantitative real-time polymerase chain reaction; RIPA = radioimmunoprecipitation assay; SC560 = 5-(4-Chlorophenyl)-1-(4-methoxyphenyl)-3-trifluoromethylpyrazol; TTBS = Tween20-tris-buffered saline; TXA_2 _= thromboxane A_2_.

## Competing interests

The authors declare that they have no competing interests.

## Authors' contributions

ESLC participated in conceiving and designing the study, performed the statistical analyses, contributed to the interpretation of the data, and edited the draft of the manuscript. HZ performed cell culture, immunoblotting, and QRT-PCR. PF performed QRT-PCR and prepared manuscript figures. SDE performed the foam cell experiments. MHP designed the SC560 and PGE experiments and assisted in interpreting the data. TP assisted HZ in executing the PG experiments. SC was instrumental in conceiving the study and critically revised the manuscript for important intellectual content. LR designed and directed the PGD_2 _experiments. ABR participated in conceiving and designing the study, supervised the study, was involved in data interpretation, and prepared the manuscript. All authors read and approved the final manuscript.
